# Causal relationship between chronic hepatitis B infection and gastric ulcer: A Mendelian randomization study

**DOI:** 10.1097/MD.0000000000044560

**Published:** 2025-09-19

**Authors:** Yiqiong Wang, Jingsi Jiang, Yutian Qin, Wenqing Wang, Jieying Li, Xinyi Li, Mamy Jayne Nelly Rajaofera

**Affiliations:** a NHC Key Laboratory of Tropical Disease Control, School of Life Sciences and Medical Technology, Hainan Medical University, Haikou Hainan, China; b School of Hainan Provincial Drug Safety Evaluation Research Center, Hainan Medical University, Haikou, Hainan, China.

**Keywords:** causal relationship, chronic hepatitis B, gastric ulcer, genome-wide association study, Mendelian randomization, single-nucleotide polymorphisms

## Abstract

Chronic hepatitis B (CHB) infection is a global health burden with various extrahepatic manifestations, but its causal relationship with gastric ulcer remains unclear. This study investigated the causal effect of CHB infection on gastric ulcer risk using Mendelian Randomization (MR). This study aimed to investigate whether CHB infection causally contributes to gastric ulcer development. We performed a 2-sample MR analysis using summary-level data from a genome-wide association study. Twenty-one single-nucleotide polymorphisms associated with CHB infection (*P* < 5 × 10^−8^, LD *r*² < 0.01) were selected as instrumental variables. Causal estimates were obtained using inverse-variance weighted (IVW) analysis, weighted median, simple mode, and MR-Egger regression. Sensitivity analyses (Cochran Q test, MR-PRESSO, leave-one-out, scatter plot, and funnel plot) assessed the robustness of results in both European and East Asian populations. The IVW analysis revealed that CHB infection significantly increased the risk of gastric ulcer (OR = 1.034, 95% CI: 1.016–1.053, *P* < .001). This association was consistently supported by the weighted median (OR = 1.035, 95% CI: 1.008–1.063, *P *= .010) and simple mode (OR = 1.049, 95% CI: 1.004–1.097, *P *= .044) method. Sensitivity analyses indicated no significant heterogeneity (Q = 12.42, *P* > .05), or horizontal pleiotropy (Egger intercept *P* = .97; MR-PRESSO global test *P =* .913). In the East Asian sample, IVW analysis produced similar findings (OR = 1.032, 95% CI: 1.012–1.053, *P* = .002). Reverse MR analysis did not support a causal effect of gastric ulcer on CHB infection. CHB infection increases the risk of gastric ulcers, emphasizing the need for considering extrahepatic manifestations in management and potential targeted interventions.

## 1. Introduction

Chronic hepatitis B (CHB) infection, caused by the hepatitis B virus (HBV), remains a significant global health burden, particularly in Asia and Africa, where it affects an estimated 250 to 350 million people.^[1]^ HBV infection is known for its ability to cause liver cirrhosis and hepatocellular carcinoma (HCC), leading to substantial morbidity and mortality worldwide.^[2,3]^ The REVEAL study and subsequent research have established a strong link between HBV DNA levels and the risk of cirrhosis and HCC, underscoring the importance of antiviral therapy in managing this condition.^[2]^ Despite these advancements, the potential extrahepatic manifestations of CHB infection are still not fully understood.

One such potential manifestation is gastric ulcer, a condition characterized by breaks in the mucosa of the stomach lining. Observational studies have reported a higher prevalence of peptic ulcers among CHB patients. For instance, 1 study noted a prevalence of 23.7% in this population, significantly exceeding that in the general population.^[4]^ Although the etiology of gastric ulcer is multifactorial, including *Helicobacter pylori* infection, nonsteroidal anti-inflammatory drug (NSAID) use, and stress,^[5,6]^ emerging evidence suggests that CHB infection may also be implicated in gastric mucosal injury.^[7]^ However, the nature of this relationship remains controversial, with some observational studies suggesting an increased incidence of gastric ulcers in patients with CHB, while others fail to find a significant association.^[4,8]^ Despite the observed association between HBV infection and an increased risk of gastric cancer, a direct causal relationship with gastric ulcers has not yet been established.^[9]^

Observational studies, however, are often limited by confounding factors and reverse causation, which hinder the ability to infer causality. Mendelian randomization (MR) offers a robust alternative by leveraging genetic variants as instrumental variables (IVs) to mimic the random allocation of alleles at conception.^[10–12]^ This method minimizes confounding and reverse causation, thereby providing insights into potential causal pathways that traditional observational studies cannot resolve.^[13]^ In the context of CHB infection and gastric ulcer, MR have the potential to shed light on the complex interplay between viral infection, immune responses, and gastrointestinal health.^[14]^ Specifically, by examining single-nucleotide polymorphisms (SNPs) associated with CHB susceptibility or progression, MR can help determine whether genetic predisposition to CHB infection influences the risk of developing gastric ulcer.

The aim of this study is to investigate the potential causal relationship between CHB infection and gastric ulcer using a MR approach. By leveraging large-scale genome-wide association study (GWAS) data and employing rigorous MR methods, a causal inference approach relying on genetic variants as IVs under assumptions of relevance, independence, and exclusion restriction, we aim to provide preliminary evidence on whether CHB infection causally contributes to the development of gastric ulcer. Our findings could significantly impact clinical practice through improved management and prevention strategies for patients with CHB. This study addresses a critical knowledge gap in understanding the potential extrahepatic manifestations of CHB infection, with results that may guide evidence-based clinical decision-making and establish new directions for future research in this actively developing field.

## 2. Materials and methods

### 2.1. Study design

**Figure 1.**
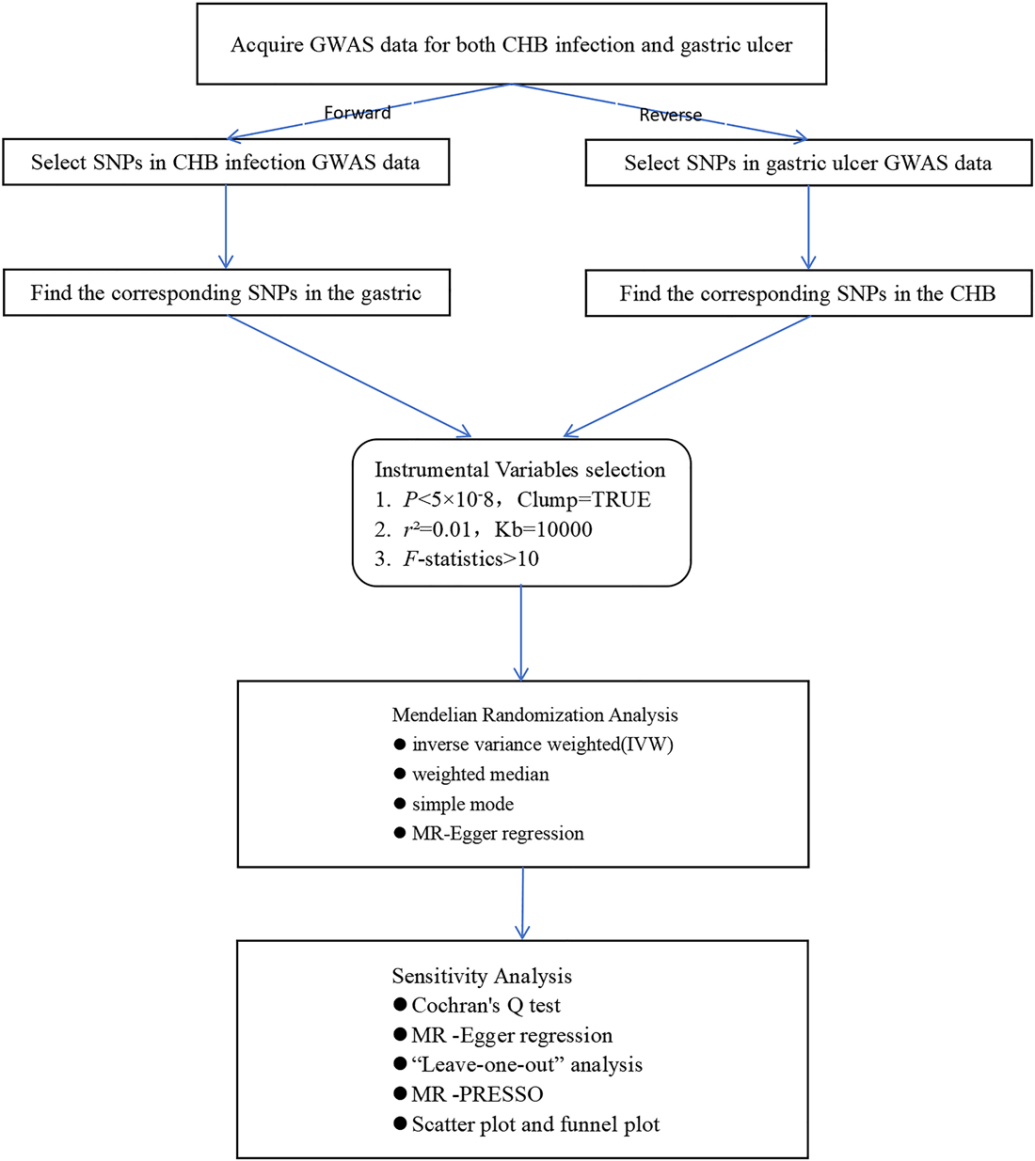
Overview of the present study design. CHB = chronic hepatitis B, MR = Mendelian randomization, GWAS = genome-wide association study, SNPs = single-nucleotide polymorphisms.

**Figure 2.**
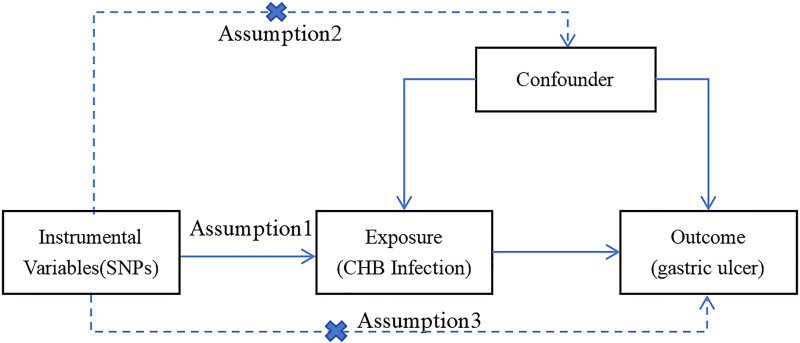
Assumptions of Mendelian randomization studies. Three hypotheses are needed to obtain a persuasive conclusion from the 2-sample MR. First, genetic instrument variables (SNPs) are directly associated with exposure (CHB infection). Second, IVs should be independent of confounders. Third, IVs affect the outcome (gastric ulcer) only through exposure (CHB infection). CHB = chronic hepatitis B, IVs = instrumental variables, SNPs = single-nucleotide polymorphisms.

In this study, MR analysis was used to assess the causal relationship between CHB infection and gastric ulcer. The framework of our study design is illustrated in Figure 1. Our bidirectional approach consisted of forward analysis, using CHB infection as the exposure factor with its significantly associated SNPs as IVs and gastric ulcer as the outcome, complemented by reverse analysis, where gastric ulcer served as the exposure factor with its associated SNPs as IVs and CHB infection as the outcome. Appropriate IVs were screened according to the 3 assumptions of MR analysis (Fig. 2): the relevance assumption: that the chosen IVs are directly associated with the exposure of interest; the independence assumption: that the chosen IVs are not associated with any confounder variables between the exposure and outcome; the exclusion restriction assumption: the chosen IVs do not affect the outcome, except through their association with the exposure.^[15,16]^

### 2.2. Data source

The CHB infection and gastric ulcer datasets were obtained from IEU OPEN GWAS (https://gwas.mrcieu.ac.uk/). As this research relied solely on aggregated public datasets, no further ethical oversight was mandated. To minimize potential bias from population stratification, we restricted our analysis to individuals of European ancestry.

Genetic association data for CHB infection were obtained from the European ancestry subset of the cross-population GWAS atlas,^[17]^ accessible via the dataset ID ebi-a-GCST90018804 (Table 1). Cases (n = 145) were defined by serologically confirmed persistent HBsAg positivity (>6 months) and exclusion of acute hepatitis or co-infection, while controls (n = 351,740) were HBsAg-negative individuals with normal liver function. Genotyping was performed using high-density arrays, followed by imputation to the 1000 Genomes Phase 3 reference panel (GRCh37). After quality control (imputation INFO > 0.8, MAF ≥ 1%), 19,079,722 SNPs were retained.

**Table 1:** Relevant information on exposure and ending.

Phenotype	Data source	GWAS	SNP	Sample size	Ethnic	Year
CHB infection	https://gwas.mrcieu.ac.uk/	ebi-a-GCST90018804	19,079,722	351,885	European	2021
Gastric ulcer	https://gwas.mrcieu.ac.uk/	ebi-a-GCST90018851	24,178,780	474,278	European	2021

CHB infection = chronic hepatitis B infection, GWAS = genome-wide association study, SNP = single-nucleotide polymorphism

Gastric ulcer genetic associations were derived from the same atlas,^[17]^ accessible via the dataset ID ebi-a-GCST90018851 (Table 1), including 6293 cases confirmed by endoscopic visualization (mucosal break ≥ 5 mm) or ICD-10 code K25.x, and 467,985 controls without gastrointestinal disease. Genotyping and imputation protocols mirrored the exposure dataset (GRCh37; INFO > 0.8, MAF ≥ 1%), yielding 24,178,780 SNPs for analysis.

### 2.3. SNP selection

To satisfy the 3 critical assumptions above, we implemented a systematic SNP selection process: applied a genome-wide significance threshold (*P *< 5 × 10^−8^) to identify SNPs strongly associated with CHB infection; pruned for linkage disequilibrium (*r*² < 0.01, genetic distance > 10000kb) to ensure independence between selected SNPs; and removed palindromic SNPs and retained only strong instruments with *F *> 10 (where *F* = [R²/(1-R²)] × [(n-k-1)/k], R²=2 × (1-MAF) × MAF×β²), eliminating weak IVs with *F *< 10.^[18]^ The reverse MR analysis followed identical methodological procedures.

### 2.4. Statistical and sensitivity analysis

The statistical analysis was conducted using the “‘Two Sample MR’” and ‘‘MR’’ packages in R software (version 4.4.2; www.r-project.org). This study was exploratory, and the analytical framework was not preregistered. All methodological decisions were documented transparently to minimize selective reporting bias. Allele mismatches were resolved by harmonizing effect alleles across datasets. Causal associations between CHB infection and risk of gastric ulcer were reported as odds ratios with 95% confidence intervals. These estimates primarily indicate the directionality of effects and are interpreted as evidence for or against a causal relationship, rather than quantifying precise clinical magnitudes.

Our primary analytical approach was the inverse-variance weighted (IVW) method, which synthesizes individual Wald estimates in a meta-analytic framework and provides reliable causal estimates when directional pleiotropy is absent.^[19,20]^ To ensure the robustness of our findings, we conducted several sensitivity analyses, including the weighted median, simple mode, and MR-Egger regression methods. The weighted median method generates consistent estimates even when a substantial proportion of genetic variants violate instrumental variable assumptions, thereby enhancing analytical robustness.^[21]^ The simple mode method facilitates straightforward comparison of differences between experimental and control groups, aiding in the interpretation of causal relationships. Finally, MR-Egger regression method was employed to evaluate horizontal pleiotropy and to detect potential confounding influences on the IVs.^[22]^ The primary causal analysis (IVW) was subjected to Bonferroni correction, with significance threshold set at α = 0.05/n, where n represents the number of independent exposures tested (α = 0.05 for a single exposure).

Heterogeneity among SNPs was assessed using Cochran Q test. A *P*-value >0.05 suggests no significant heterogeneity for SNPs strongly associated with CHB infection.^[23]^ Horizontal pleiotropy was evaluated using the intercept from MR-Egger regression, where a *P*-value >0.05 indicates the absence of horizontal pleiotropy for SNPs associated with CHB infection. Additionally, the MR-PRESSO method was applied to detect horizontal pleiotropy through a global test and to identify potential outliers using an outlier test.^[24]^ The leave-one-out analysis, each instrumental variable was excluded one at a time, with no significant changes in the results, was used to confirm the robustness of the findings. Scatter plot and funnel plot were also used to assess the stability of the results. Moreover, the study analyzed data from East Asian populations under similar conditions, utilizing datasets for CHB infection (ID: ebi-a-GCST90018584) and gastric ulcer (ID: ebi-a-GCST90018631).

### 2.5. Ethical review

Ethical approval was not required for this study as it involved only analysis of publicly available GWAS summary statistics from the IEU OPEN GWAS database without access to individual participant data. Institutional review board approval was waived per international guidelines for de-identified genomic analyses.

## 3. Results

### 3.1. Causal effect of CHB infection on gastric ulcer

In this study, we identified 21 SNPs (*P* < 5 × 10^−8^, LD *r*² < 0.01) significantly associated with CHB infection, all of which exhibited robust strength with *F*-values > 10, indicating absence of weak instrument bias (Table 2). After harmonization, all 21 SNPs extracted for both exposure and outcome showed consistent directions of effect, ensuring proper alignment for the MR analysis. The primary IVW analysis revealed that CHB infection significantly increased the risk of gastric ulcer (OR = 1.034, 95% CI: 1.016–1.053, *P *< .001). This finding was corroborated by weighted median analysis (OR = 1.035, 95% CI: 1.008–1.063, *P* = .010) and simple mode analysis (OR = 1.049,95% CI: 1.004–1.097, *P* = .044). The MR-Egger regression showed a consistent effect direction, though with wider confidence intervals (OR = 1.033, 95% CI: 0.973–1.097, *P* = .300) (Table 3). Notably, while the MR-Egger regression analysis yielded a *P*-value exceeding the conventional significance threshold, all 4 MR analytical approaches produced positive beta coefficients, demonstrating consistent directional effects. The MR-Egger regression incorporates an intercept term, distinguishing it from the IVW approach. In the absence of significant heterogeneity and horizontal pleiotropy, the IVW method typically provides more precise estimates than MR-Egger regression.^[21]^ Therefore, we prioritized the IVW results in our interpretation. Overall, our MR analysis provides statistically significant evidence supporting a causal relationship between CHB infection and gastric ulcer risk.

**Table 2:** General information on the instrumental variables.

SNP	EA	OA	*F*	Related to chronic hepatitis B infection			Related to gastric ulcer		
				b	se	*P*	b	se	*P*
rs114484678	C	T	40.15368513	−0.3143	0.0496	2.33201 × 10^−10^	−0.0024	0.019	.899
rs115552552	T	C	50.55373868	−0.5553	0.0781	1.12902 × 10^−12^	−0.0415	0.0269	.123
rs115888238	C	G	80.96697584	−0.4904	0.0545	2.1732 × 10^−19^	−0.0167	0.0194	.388
rs11754012	T	C	77.13695773	−0.4892	0.0557	1.61585 × 10^−18^	−0.0308	0.0174	.077
rs117810449	C	T	46.10613557	0.3171	0.0467	1.15505 × 10^−11^	0.0146	0.0194	.451
rs12660083	T	C	34.78785875	0.22	0.0373	3.66598 × 10^−09^	0.0154	0.0154	.317
rs16870693	A	C	43.0033284	0.2728	0.0416	5.54753 × 10^−11^	−0.0036	0.0175	.837
rs28715762	C	A	67.92217532	−0.5085	0.0617	1.6531 × 10^−16^	0.0123	0.025	.622
rs28746784	T	C	143.226847	0.4452	0.0372	6.05202 × 10^−33^	0.0004	0.0158	.981
rs28826696	C	T	181.9040105	0.526	0.039	1.50314 × 10^−41^	0.0107	0.0163	.513
rs3094196	G	A	43.66531274	0.1989	0.0301	3.83707 × 10^−11^	0.0104	0.0115	.364
rs3130779	G	A	58.3336526	0.3246	0.0425	2.29615 × 10^−14^	0.0268	0.0141	.058
rs34975158	A	G	63.43160818	−0.5145	0.0646	1.72982 × 10^−15^	−0.0197	0.0227	.385
rs3998115	C	G	71.10937641	0.4444	0.0527	3.53916 × 10^−17^	0.0042	0.0148	.778
rs549060653	T	C	47.47824415	−0.5409	0.0785	5.56032 × 10^−12^	−0.0304	0.0304	.317
rs6913309	A	T	117.3216056	−0.495	0.0457	2.52872 × 10^−27^	−0.0176	0.0146	.227
rs73403026	A	G	34.72310693	0.2799	0.0475	3.84601 × 10^−9^	0.025	0.0183	.171
rs73739611	T	C	192.307775	0.4396	0.0317	8.20729 × 10^−44^	0.0058	0.0132	.660
rs7741871	A	G	56.83326521	−0.3596	0.0477	4.75773 × 10^−14^	−0.0339	0.0148	.022
rs79690458	T	A	60.43808533	−0.5302	0.0682	7.43875 × 10^−15^	−0.027	0.0234	.248
rs9277665	A	G	41.85934098	−0.2148	0.0332	1.00501 × 10^−10^	0.0104	0.0125	.405

b = beta, EA = effect allele, F = *F*-statistic, OA = other allele, p = *P*-value, se = standard error, SNP = single-nucleotide polymorphism.

**Table 3:** Results of the MR study on causal association between CHB infection and gastric ulcer (European).

Method	nsnp	beta	se	*P*	or	or_lci95	or_uci95	Heterogeneity	Pleiotropy test
IVW	21	0.034	0.009	0.0003	1.034	1.016	1.053	Q = 12.424, Q_pval = 0.901	
Weighted media	21	0.035	0.013	0.010	1.035	1.008	1.063		
Simple mode	21	0.049	0.023	0.044	1.050	1.004	1.097		
MR-Egger regression	21	0.033	0.030	0.300	1.033	0.973	1.097	Q = 12.423, Q_pval = 0.867	egger_intercept = 0.0004, Pval = 0.97

IVW = inverse-variance weighted, nsnp = number of single-nucleotide polymorphisms, or = odds ratio, *p* = *p*-value, se = standard error

**Figure 3.**
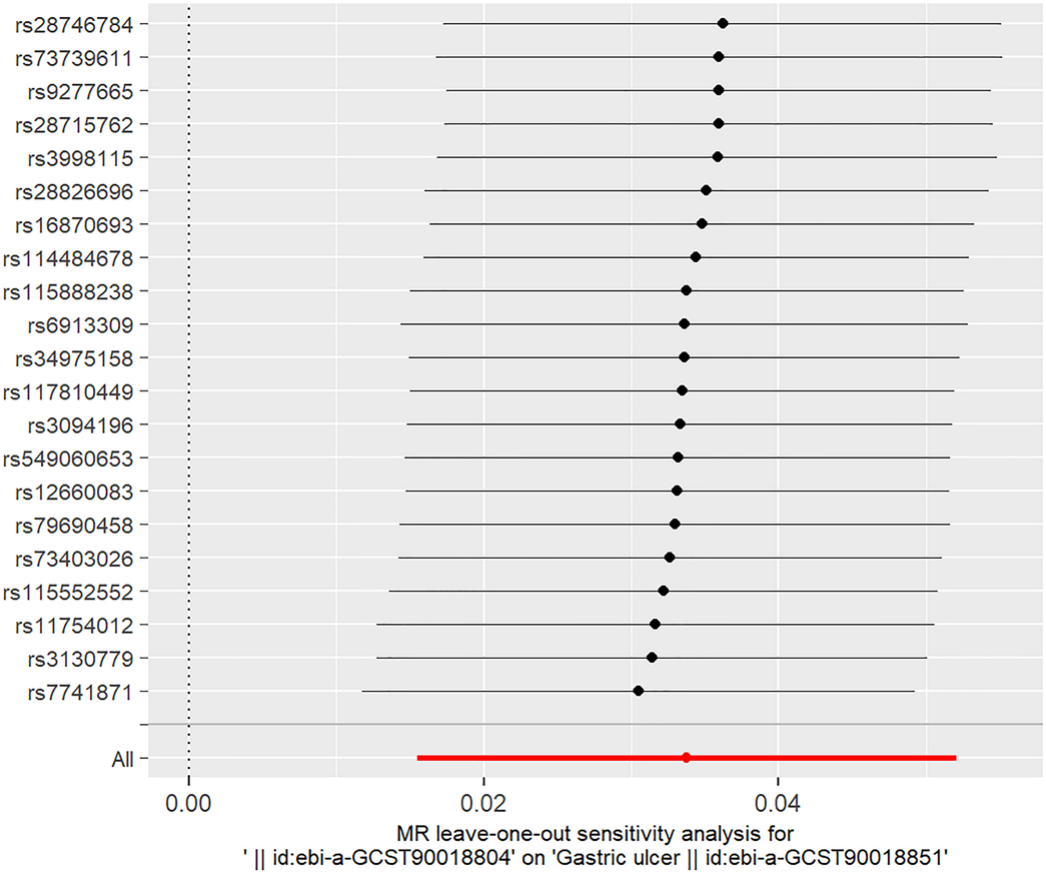
Conduct a leave-one-out sensitivity analysis to assess the effect of CHB infection on gastric ulcer using MR. This analysis involves sequentially removing 1 IV at a time and recalculating the MR results with the remaining IVs. The analysis focuses on the European population. CHB = chronic hepatitis B, IV = instrumental variable, MR = Mendelian randomization

**Figure 4.**
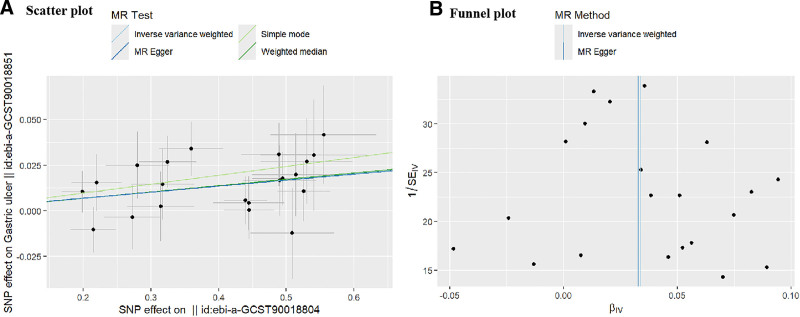
(A) Scatter plot illustrating the causality between CHB infection and gastric ulcer. Each line represents the estimated MR effect across different models, while the plot displays the distribution of individual effect estimates. (B) Funnel plot assessing pleiotropy in the causal associations between chronic hepatitis B infection and gastric ulcer. CHB = chronic hepatitis B.

The Cochran Q test revealed no significant heterogeneity (Q = 12.42, *P* > .05), and the pleiotropy assessment (Egger intercept = 0.0004, *P* = .97) indicated absence of horizontal pleiotropy (Table 3). Additionally, The MR-PRESSO analysis did not identify any outliers, as evidenced by the Global Test’s *P*-value of 0.913, which is well above the conventional threshold for statistical significance. This result suggests that there is no substantial horizontal pleiotropy present in our analysis. Leave-one-out sensitivity analyses demonstrated robust causal estimates, with no significant alterations in MR results following sequential removal of individual SNPs strongly associated with CHB infection (Fig. 3). Scatter plot confirmed that results were not influenced by outliers (Fig. 4A), while funnel plot analysis suggested minimal impact of potential confounding factors on the MR estimates (Fig. 4B). Importantly, a parallel analysis in an East Asian population sample yielded consistent results, showing a significantly increased risk of gastric ulcer associated with CHB infection (IVW: OR = 1.032, 95% CI: 1.012–1.053, *P* = .002) (Table 4).

**Table 4:** Results of the MR study on causal association between CHB infection and gastric ulcer (East Asian).

Method	nsnp	beta	se	*P*	or	or_lci95	or_uci95
IVW	21	0.032	0.010	0.002	1.032	1.012	1.053
Weighted median	21	0.020	0.014	0.151	1.020	0.993	1.048
Simple mode	21	0.008	0.024	0.726	1.008	0.963	1.056
MR-egger regression	21	0.026	0.046	0.577	1.026	0.938	1.123

CHB = chronic hepatitis B, IVW = inverse-variance weighted, nsnp = number of single-nucleotide polymorphisms, or = odds ratio, *p* = *p*-value, se = standard error.

### 3.2. Causal effect of gastric ulcer on CHB infection

After removing linkage disequilibrium, only 2 SNPs (rs2976387, rs12675227) remained as valid instruments for gastric ulcer. The IVW analysis did not reveal a significant causal relationship between gastric ulcer and CHB infection (OR = 1.184, 95% CI: 0.760–1.845, *P* = .450). However, the limited number of available IVs restricted the reliability of sensitivity analyses for this reverse causation assessment.

## 4. Discussion

The present MR study investigated the potential causal relationship between CHB infection and gastric ulcer risk. Despite the intricate interplay between viral hepatitis and various gastrointestinal disorders, the specific link between CHB infection and gastric ulcer has been less explored.^[25]^ By leveraging genetic variants as IVs, our approach mitigated confounding factors and reverse causation that often compromise traditional observational studies. Our primary analysis using the IVW method demonstrated a statistically significant association between CHB infection and an increased risk of gastric ulcer. This association was supported by the weighted median and simple mode analyses, consistently indicated a positive effect direction.

To evaluate reverse causation, we performed reverse MR using 2 valid SNPs (rs2976387, rs12675227) as instruments for gastric ulcer. The IVW analysis indicated no significant causal effect (OR = 1.184, 95% CI: 0.760–1.845, *P* = .450). While this nonsignificant finding aligns with our primary causal direction, interpretation is severely limited by having only 2 instruments: power to detect plausible effects is extremely low, sensitivity analyses for pleiotropy could not be performed, and estimates are highly vulnerable to outlier bias. Therefore, this analysis cannot reliably exclude a true causal effect of gastric ulcer on CHB infection or definitively rule out reverse causation.

Our results align with some previous observational studies that have hinted at a potential link between CHB infection and gastric ulcer, albeit with mixed conclusions.^[9,26,27]^ A potential mechanism linking CHB infection to an increased risk of gastric ulcer may involve a complex interplay of chronic inflammation, immune dysregulation, and oxidative stress.^[28]^ Persistent HBV infection is known to induce systemic inflammatory responses, which could disrupt the gastric mucosal barrier and impair its regenerative capacity.^[29]^ Inflammatory cytokines and oxidative stress generated by chronic hepatitis may weaken the integrity of the gastric epithelium, rendering it more susceptible to damage by other ulcerogenic factors such as Helicobacter pylori infection or NSAID use.^[30]^ Additionally, CHB-related immune alterations might modify the gastric microbiota composition, further contributing to mucosal injury.^[30,31]^ The consistency of our findings with biological mechanisms underlying CHB and gastric ulcer strengthens the credibility of our results. Furthermore, the use of MR, which leverages genetic variation to infer causality, minimizes the influence of confounding factors and reverse causation, thus enhancing the robustness of our conclusions.^[32]^

Despite the strengths of our MR approach, several limitations of our study should be acknowledged. First, the MR-Egger regression analysis results suggested a lack of robust evidence for a causal effect, likely due to insufficient statistical power or the presence of residual pleiotropy.^[33]^ This limitation highlights the need for caution in interpreting the findings and underscore the importance of larger sample sizes and more robust genetic instruments to validate the causal relationship. Second, our analyses revealed some discrepancies between European and East Asian populations. Although the IVW estimates were statistically significant in both groups, the weighted median and simple mode methods did not reach significance in the East Asian sample, suggesting possible genetic heterogeneity, differences in instrument validity, or population-specific environmental factors that could influence the causal estimates.^[33]^ In addition, population-specific environmental factors, lifestyle differences, or disease epidemiology may also affect the accuracy of causal estimates.^[34,35]^ Third, the MR analysis assumes a linear relationship between CHB infection and gastric ulcer risk, which may not fully capture potential nonlinear or threshold effects.^[36]^ Additionally, developmental compensation and canalization processes may modulate gene expression over a lifetime, potentially attenuating the impact of genetic variants on CHB infection and biasing causal estimates.^[37,38]^ The relatively modest number of SNPs used as IVs (n = 21) may also limit the precision and robustness of our estimates. Finally, while our analysis was restricted to individuals of European ancestry (with a parallel analysis in an East Asian cohort), the generalizability of our findings to other populations remains to be determined.

Despite these limitations, our study provides preliminary evidence suggesting a causal relationship between CHB infection and the risk of gastric ulcer. These findings have important implications for clinical practice and public health strategies, particularly in regions where CHB is endemic and gastric ulcer is a common gastrointestinal disorder. If these findings are confirmed in larger validation studies, they will have a significant clinical impact. The identification of CHB infection as a causative factor for gastric ulcers may reduce the incidence of gastric ulcers and related complications by providing more aggressive antiviral therapy for patients with CHB infection.^[39]^ In addition, screening patients with CHB infection for gastric ulcers may become the standard of clinical management for early detection and treatment.^[28]^

Future research should aim to replicate our findings in larger samples, ideally through GWAS meta-analyses, to enhance statistical power and generalizability.^[40]^ Additionally, longitudinal studies and animal models could provide further insights into the biological mechanisms underlying this association.^[41]^ It is also important to note that our study focused on the genetic level of causality and did not explore other potential pathways through which CHB might influence the risk of gastric ulcer. Therefore, future research should consider a multifaceted approach, incorporating epidemiological, clinical, and biological data, to comprehensively understand the relationship between CHB and gastric ulcer.^[42,43]^

## 5. Conclusion

This MR study provides preliminary evidence suggesting a causal relationship between CHB infection and gastric ulcer risk. While the findings are promising, further research is needed to validate these results and explore the underlying biological mechanisms in larger population samples.

## Acknowledgments

The authors thank Youxin Wang for his valuable contributions to this study. The authors also express their gratitude to the participants and investigators of the study.

This study utilized publicly available genome-wide association study (GWAS) summary statistics from the IEU OPEN GWAS database (https://gwas.mrcieu.ac.uk/). All datasets were fully anonymized, and no individual-level data were accessed. Ethical approval for the original GWAS studies was obtained by the respective data contributors, and this secondary analysis of de-identified data does not require additional ethical review. The authors sincerely thank related investigators for sharing the GWAS summary statistics included in this study.

## Author contributions

**Conceptualization:** Jingsi Jiang.

**Data curation:** Yiqiong Wang, Yutian Qin, Wenqing Wang, Xinyi Li.

**Formal analysis:** Yiqiong Wang.

**Funding acquisition:** Jingsi Jiang, Jieying Li.

**Resources:** Jingsi Jiang.

**Software:** Yiqiong Wang.

**Supervision:** Mamy Jayne Nelly Rajaofera.

**Validation:** Mamy Jayne Nelly Rajaofera.

**Visualization:** Jingsi Jiang.

**Writing – original draft:** Yiqiong Wang.

**Writing – review & editing:** Mamy Jayne Nelly Rajaofera.
